# Next-generation Solutions: Are Patients Ready for Electronic Artificial Urinary Sphincters for Male Incontinence?

**DOI:** 10.1016/j.euros.2025.02.004

**Published:** 2025-02-21

**Authors:** Marc Kidess, Troya Ivanova, Julian Hermans, Leo Stadelmeier, Marina Hoffmann, Nikolaos Pyrgidis, Julian Marcon, Michael Chaloupka, Ricarda M. Bauer, Christian G. Stief, Yannic Volz

**Affiliations:** a Department of Urology, University Hospital of Munich, Munich, Germany; b Urologie Maximilianstrasse, Munich, Germany

**Keywords:** Incontinence, Artificial male sphincter, Technology

## Abstract

**Background and objective:**

Urology is characterized by continuous innovation. The inception of robot-assisted radical prostatectomy (RP) marked a pivotal technological advance and further advances in digital treatment options for stress urinary incontinence (SUI) are emerging. Our aim was to assess patient willingness to receive an electronic artificial urinary sphincter (eAUS) implant and identify associated concerns.

**Methods:**

Patients who received a first AUS implant (AMS800 system) for post-RP SUI from March 2013 to December 2023 were included. An anonymous survey was used to collect data on demographics, current AUS satisfaction, daily technology use, interest in an eAUS, and concerns about potential eAUS technical malfunctions. Data were analyzed using SPSS, with significance set at *p* < 0.05.

**Key findings and limitations:**

Out of 345 patients, 208 (60.2%) completed the questionnaire. The majority were aged 71–80 yr (51.7%) and had a university education (37.7%). Satisfaction with their AUS was high: 79.8% of the respondents were satisfied, 88.9% were satisfied with its handling, and 89.4% would choose an AUS implant again. Notably, 60.4% showed interest in an eAUS, with younger respondents and those who use technology on a daily basis expressing greater interest. Preferred control methods included remote-based (78.4%) and smartphone-based (60.0%) options. Concerns about system malfunction (66.4%), connection loss (65.9%), and battery issues (60.0%) were prevalent.

**Conclusions and clinical implications:**

There was significant patient interest in an eAUS in our survey, especially among younger individuals and those who use technology daily. Despite high satisfaction with current AUS devices, addressing potential technical malfunctions and patient concerns is crucial for broader acceptance of an eAUS. Patient concerns about technological malfunctions seem to outweigh worries about medical issues.

**Patient summary:**

Urology is becoming more advanced with technologies like robotic surgery and electronic artificial urinary sphincters (eAUS). According to our survey, most patients are happy with their current sphincters and are open to eAUS, especially younger patients who are familiar with technology. However, patients are concerned about system malfunctions and connection loss. More research is needed to address technical issues and patient concerns.

## Take Home Message

The majority of patients who responded to our survey are satisfied with their current artificial urinary sphincter (AUS). There was a high level of interest in an electronic AUS, especially among younger patients with an affinity for technology.

## 1 Introduction

Urology is a discipline of innovation. In 2000, the first robot-assisted radical prostatectomy (RARP) was performed [1], marking the beginning of a technical revolution in urological surgery, with RARP accounting for 85% of RP procedures in the USA by 2013 [2]. There is also ongoing digitalization in urology, including new perioperative imaging technologies such as superimposed magnetic resonance imaging–based three-dimensional models of the prostate on the video display of robotic surgical systems [3], [4].

Digitalization and technology are also yielding advances for stress urinary incontinence (SUI), a condition affecting up to 20% of patients after RP despite conservative treatments such as physiotherapy [5], [6]. After failure of conservative measures, surgical solutions are an important part of the treatment armamentarium for SUI. For male patients with severe SUI, the European Association of Urology guidelines recommend implantation of an artificial urinary sphincter (AUS) [7]. These systems show cure rates up to 90% [8]. Since the introduction of the first AUS in the 1970s by Dr. Brantley Scott, these devices have been modified several times [9], [10], [11]. In 2023, UroMems, a French company founded in 2011 to develop innovative solutions for SUI treatment, announced the first ever implantation of UroActive, an automated electronic AUS (eAUS), in a patient with promising results [12]. The UroActive myoelectromechanical system allows occlusive pressure on the urethra to vary instantly based on patient activity. Activation and manual pressure regulation can be performed wirelessly, such as via a smartphone, and a remote control enables the patient to void [13]. A recent study demonstrated that UroActive implantation is feasible in men [14]. Introduction of eAUS devices has the potential to improve AUS handling because of greater user friendliness for patients and medical staff. Complications such as urethral injury frequently result from catheterization performed without proper deactivation of the device, often necessitating AUS explantation. In addition, pump migration can cause discomfort or impairment of device function, in the worst case leading to complications such as retention or renal failure [15]. eAUS devices could mitigate such complications by offering more user-friendly handling. Another advantage is the possibility of adjustment of the cuff closing pressure [16]. This could support continence in stressful situations (eg, during sports activities) and might reduce the risk of urethral erosion.

It seems that innovation in urological therapies extends beyond the operating theater to the treatment of SUI and that current treatment strategies now face a digital and technical revolution. However, there have been no studies on patient preferences and needs regarding eAUS devices. Consequently, the aim of this study was to determine if patients suffering from SUI would be interested in an eAUS implant to reduce their incontinence and to identify potential concerns about this new technology.

## 2 Patients and methods

### 2.1 Patient population

Patients who received a first AUS implant for treatment of severe post-RP SUI between March 2023 and December 2023 in our tertiary referral center were included in the study. Patients who received a second implant or exchange of the whole system or parts were excluded. The AMS 800 system (AMS/Boston Scientific, Marlborough, MA, USA) was the sole implant used, and the procedure was performed using the perineal approach previously described at our institution [17]. All patients were treated by two surgeons.

### 2.2 Creation of the questionnaire

An anonymous questionnaire consisting of 14 questions was developed (Supplementary material) by a group of three urologists with extensive expertise in incontinence treatment. General questions addressed the patient’s age, highest level of education, and previous medical conditions. Specific questions on the current AUS implant included the time since implantation, the patient’s satisfaction with its handling, and whether the patient would choose to undergo the procedure again. The questionnaire also evaluated daily use of technologies such as smartwatches and smartphones.

Patients were also asked about their interest in implantation of an eAUS, their preferred method of operation for this device, and whether they would like to be able to adjust the sphincter closing pressure according to their activity level. In addition, there were questions on concerns regarding technical malfunctions or medical complications related to an eAUS. An English translation of the questionnaire is provided in the Supplementary material. As this is the first study investigating patient interest in an eAUS, validation of the questionnaire was not possible. No pretesting of the questionnaire was performed.

All patients received a letter containing the questionnaire and a specific QR code that enabled them to complete the questionnaire online. Patients who chose to respond on paper could return their answers free of charge. The invitation letter informed patients that the eAUS is still in the developmental stage and that its long-term function and safety have not yet been investigated. It was assumed that the long-term functional results would be the same with the eAUS as with a conventional AUS. The study and the questionnaire were approved by the local ethics committee of LMU University Hospital (reference 24-0131 KB).

### 2.3 Statistical analysis

Statistical analyses, reporting, and interpretation of the results were conducted in accordance with the guidelines for reporting of statistics for clinical research in urology [18]. Statistical analysis was performed using SPSS v29 (IBM, Armonk, NY, USA). The Shapiro-Wilk test was used to assess whether variables followed a normal distribution. Descriptive statistics are reported as the median and mean for continuous variables, and the frequency and proportion for categorical variables. Binary logistic regression analysis was used to determine the factors influencing patient openness to an eAUS system. Adjustments were made for confounding factors such as age, education, and technological interest. A two-sided *p* value of <0.05 was considered statistically significant.

## 3 Results

### 3.1 Baseline characteristics

**Fig. 1:**
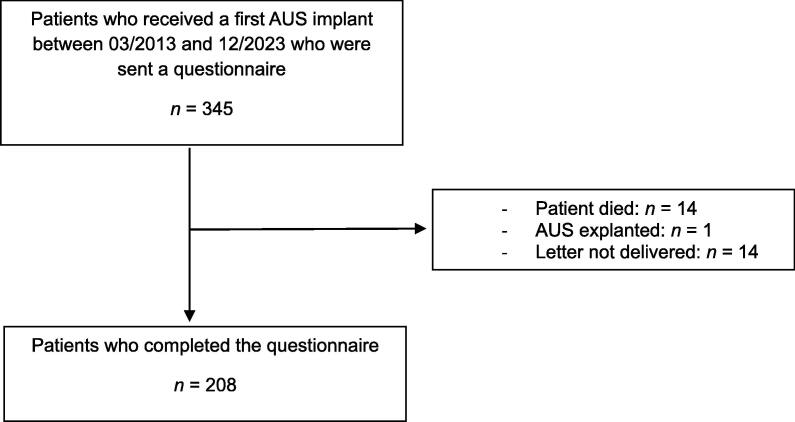
Study flowchart. The questionnaire was sent to 345 patients who received an artificial urinary sphincter (AUS) implant between March 2013 and December 2023, of whom 208 completed the questionnaire. For one patient, the AUS was explanted, 14 patients died, and the letter was not delivered because of a change in address for another 14 patients.

Overall, 345 patients received an AUS implant between March 2013 and December 2023. Of these, 208 patients (60.2%) participated in the study and completed the questionnaire. Fourteen patients (4.1%) had died and another 14 (4.1%) did not receive the letter because of a change in address (Fig. 1).

Baseline characteristics are listed in Table 1. A total of 36 participants (17.3%) completed the questionnaire online (Table 2). The majority of the participants were aged 71–80 yr (107/208; 51.7%) and had completed either university education (78/208; 37.7%) or vocational training (74/208; 35.7%). The most prevalent comorbidities were oncological (172/208; 83.1%) and cardiovascular diseases (96/208; 46.4%) and diabetes (39/208; 18.8%). In most cases, the AUS implant was received 6–10 yr before the survey (69/208; 33.3%).

**Table 1:** Baseline characteristics

Parameter	Patients, *n* (%)
Age group (yr)	
50–60	9 (4.3)
61–70	33 (15.9)
71–80	107 (51.7)
81–90	58 (28.0)
Highest educational level	
Secondary school certificate	24 (11.6)
Higher school certificate	3 (1.4)
Vocational training	74 (35.7)
University education	78 (37.7)
PhD thesis	14 (6.8)
No answer	15 (7.0)
Comorbidities	
Cardiac comorbidities	96 (46.4)
Neurological comorbidities	25 (12.1)
Diabetes	39 (18.8)
Musculoskeletal comorbidities	34 (16.4)
Oncological comorbidities	172 (83.1)
Other	21 (10.1)
None	9 (4.3)
Online completion of the questionnaire	36 (17.3)
How long ago was your sphincter implanted? (yr)	
<1	24 (11.6)
1–2	36 (17.4)
3–5	64 (30.9)
6–10	69 (33.3)
>10	12 (5.8)

**Table 2:** Survey results on demand for an electronic artificial urinary sphincter

Question			Response, *n* (%)
Would you be open to receive an electronic artificial urinary sphincter?			
Yes			125 (60.4)
No			73 (35.3)
No answer			9 (4.3)
If yes, how would you like to control your electronic artificial urinary sphincter?			
App-based on a smartphone			75 (60.0)
App-based on a smartwatch			34 (27.2)
Remote-based			98 (78.4)
Would you like to be able to change sphincter closing pressure in relation to the situation (eg, sport/sleep)?			
Yes			115 (92.0)
**Category**	**Interested in an electronic artificial sphincter,***n* (%)		*p* value
	No	Yes	

Age group (yr)			**0.006**
50–60	0 (0.0)	9 (100.0)	
61–70	7 (21.2)	26 (78.8)	
71–80	39 (38.6)	62 (61.4)	
81–90	27 (49.1)	28 (50.9)	
Currently satisfied	59 (35.5)	107 (64.5)	0.457
Would choose again	64 (34.4)	122 (65.6)	**0.002**
Happy with handling	66 (35.7)	119 (64.3)	0.189
Online questionnaire	3 (8.8)	31 (91.2)	**<0.001**
Daily technology use			
Smartphone	43 (29.5)	103 (70.5)	**<0.001**
Tablet	10 (17.9)	46 (82.1)	**<0.001**
Smartwatch	3 (15.8)	16 (84.2)	0.051
Computer	37 (29.6)	88 (70.4)	**0.010**
None	8 (47.1)	9 (52.9)	0.331
Any technology	55 (32.9)	112 (67.1)	**0.008**

Overall, 166 patients (79.8%) were currently satisfied with their AUS. In addition, 185 patients (88.9%) were satisfied with the handling of their AUS and 186 (89.4%) would opt for an AUS implant again (Table 2).

### 3.2 Interest in an eAUS

A total of 125 patients (60.4%) expressed interest in an eAUS. Interest in the eAUS varied significantly across age groups (*p* = 0.06), with younger age groups reporting higher interest. Notably, the group of participants who answered the questionnaire online had a higher eAUS interest rate (*p* < 0.001). Daily use of various technologies also correlated with interest in an eAUS (*p* < 0.008). Users of technology, especially those using a smartphone (*p* < 0.001), tablet (*p* < 0.001), or computer (*p* < 0.01), were significantly more interested in implantation of an eAUS. Concerning eAUS handling and operation, the majority of participants (78.4%) would prefer a remote-based control system, followed by app-based control via a smartphone (60.0%). In addition, 115 participants (92.0%) would value ability to adjust the eAUS closing pressure according to their activity level. These and further results are presented in Table 2.

There were no significant differences in interest in an eAUS on stratification by pre-existing medical conditions (cardiovascular diseases *p* = 0.15; neurological diseases *p* = 0.22; diabetes mellitus *p* = 0.55; musculoskeletal diseases *p* = 0.94; oncological diseases *p* = 0.47; other diseases *p* = 0.28).

### 3.3 Concerns regarding eAUS complications

**Fig. 2:**
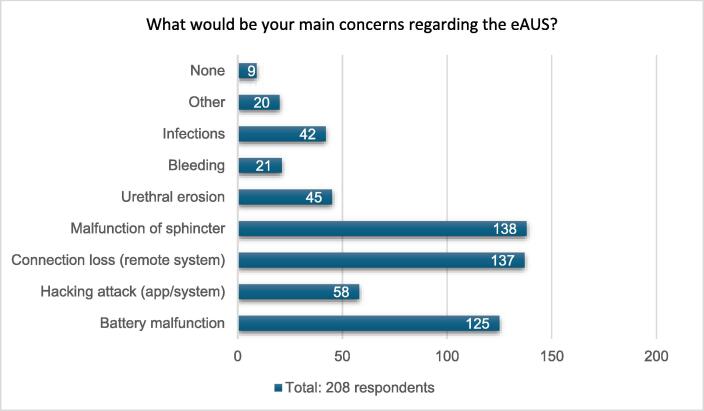
Participant concerns regarding an electronic artificial urinary sphincter (eAUS).

Regarding potential eAUS complications, the majority of participants expressed concern about potential malfunction of the system (138/208; 66.4%), loss of connection between the remote control and the eAUS (137/208; 65.9%), and battery malfunction (125/208; 60.0%). By contrast, only a few participants expressed concern about medical complications such as infection and urethral erosion (Fig. 2).

## 4 Discussion

Since the advent of robotic surgery, urology has been experiencing a technical and digital revolution. Introduction of an AUS in the early 1970s represented a major advance in the treatment of SUI, and this device has now been used in clinical practice for more than 50 yr. Recent innovations, such as adjustable cuffs, have further refined this technology [19]. The next anticipated advance is the introduction of an eAUS. However, it is not known whether this innovation for the treatment of SUI will be accepted or desired by patients. Therefore, we conducted a single-center investigation to assess the interest in an eAUS among patients with post-RP SUI who had already undergone implantation of an established AUS system.

Our results show that nearly 80% of the survey respondents are currently satisfied with their AUS, and nearly 90% are happy with the current handling of their AUS and would opt for implantation again. Similar rates of satisfaction were reported in different studies (between 19% and 45% of patients were satisfied, and between 28% and 58% were very satisfied) [20], [21]. The data indicating that a low proportion of patients are unsatisfied with their AUS may explain our response rate of 60%, as patients who are satisfied may have been less inclined to complete the questionnaire as a means to make their dissatisfaction known. By contrast, the high eAUS interest across all age groups may explain why the response rate was as high as 60%.

It is important to note that we assumed that an eAUS would perform similarly to the current AUS in terms of both incontinence control and complication rates. Patients were informed that actual outcomes remain unknown, as the eAUS is not yet in routine clinical use, and no comparative studies between the eAUS and the current AUS are available. Should the results of the eAUS prove to be either superior or inferior, patient interest in the device may vary accordingly.

The vast majority of our survey respondents were aged >70 yr. Although all age groups expressed interest in eAUS implantation, subgroup analysis revealed that especially younger patients. This is in line with other studies showing that in the older population, younger individuals are more likely to engage with new technologies such as smartphones [22], [23].

Previous studies have explored modifications to existing AUS systems (eg, AMS 800) that involved integration of electromagnetism or remote controls [11]. The first procedures to implant an eAUS (UroActive) in six male patients (median age 69 yr) who were incontinent after RP have shown promising results [14], [24]. No malfunctions or complications have been reported so far. Given that system malfunction was the most frequent concern among our study participants, further investigations into this issue are necessary. At 3 mo after activation of the UroActive device, patients averaged approximately 8 micturitions per day and spent 7 h each day with low-pressure settings. Furthermore, patients experienced an increase in continence rate of >50% on a 24-h pad test. The authors concluded that the UroActive device met the safety and efficacy criteria after 3 mo, thus allowing them to continue the study [24].

If eAUS implants emerge as a new treatment option for male SUI in the future, careful selection of suitable patients will be necessary, and the needs and concerns of individual patients should be addressed. Our study demonstrated that patients with higher technical affinity and use are more likely to be open to an eAUS implant. This finding aligns with research indicating that the adoption of new technologies is often related to the knowledge and skills of potential users [25]. Furthermore, no significant differences in preferences were observed in subgroups with different comorbidities and pre-existing conditions. This factor does not appear to influence the interest in new technology. In a study by Korchut et al [26], patients with neurological diseases such as multiple sclerosis reported that technical was very helpful in managing their daily lives. A possible explanation for our observation is that our study exclusively involved patients who already had an AUS implant and had sufficient manual skills at the time of implantation. If patients without prior AUS experience were asked the same question, pre-existing comorbidities might have a greater influence on their interest in the new technology.

As the majority of our respondents identified potential malfunction of the system, loss of connection between the remote control and eAUS, and battery malfunction as concerns, these issues must be considered during eAUS development. The device should incorporate options for manual operation without reliance on electricity, along with noninvasive charging methods or long-lasting batteries. Furthermore, rigorous reliability testing is essential to ensure patient confidence in the eAUS system.

Interestingly, we observed that most participants would prefer a remote control for eAUS operation rather than an app on their smartphone or smartwatch. Consistent with previous studies, a smartphone was the technology most frequently used by older people [22]. One potential reason may be concern about loss of connection between the smartphone and the eAUS or a fear of system hacking that can occur with smartphones [27]. Previous research demonstrated that older people do not use new technologies such as the internet because they feel that new technologies are too complex and too insecure [23]. The 2015 Austrian mobi.senior.A study investigated the use of smartphones and tablets by older people. The results showed while easy use of apps is important for acceptance, most apps had usability issues because of their complexity [28]. Challenges such as use of a touchscreen, fear of damaging the device, and concerns about incurring costs because of misuse have been noted [29]. As older individuals are probably more familiar with the use of remote controls for devices such as televisions, the perceived simplicity of remote controls may explain their preference in the current study. Nevertheless, a 2011 study showed that older adults represent an excellent market for disruptive innovations, as this group has an increasing demand for affordable, user-friendly products and new services [30], [31]. This may explain why older participants also expressed an interest in eAUS. Rodler et al [3] found that urological patients are willing and open to trying digital therapeutics.

In the future it will be essential to identify suitable candidates for eAUS implantation. Our results indicate that while a majority of our respondents were interested in eAUS implantation, the interest was particularly high among frequent users of technology, younger patients, and those with a higher level of physical activity. Pre-existing medical conditions or educational background did not seem to have any influence on eAUS interest. It is necessary to address the concerns identified by our respondents. Finally, further investigation is needed on how to prevent malfunctions and develop strategies to address these problems to provide maximum safety for patients.

Our study is not devoid of limitations. First, we were not able to use a validated questionnaire as this is the first study to evaluate the interest in an eAUS. Despite being the only survey to date on eAUS interest, with a high response rate, the study primarily reflects general interest and does not provide a detailed assessment of individual patient needs. Another limitation is the single-center setting. Furthermore, as the response rate was not 100%, we cannot fully determine the urgency of the eAUS demand. Presupposing that long-term eAUS functional results and complications could be the same as with conventional devices could also explain the responses. A multicenter study involving centers in different countries could help in gaining a better picture of patients needs and wishes regarding eAUS. However, we believe that our response rate of 60% shows the significance and relevance of this topic and the need to address eAUS options in the near future.

## 5 Conclusions

Urology is experiencing a transformative shift towards digitalization and technological innovation, extending from robot-assisted surgeries to the development of an eAUS. Our study demonstrates that a substantial majority of patients are satisfied with their current AUS and are open to the potential benefits of an eAUS, with younger and technologically adept patients showing particularly high interest. There is high patient interest in an eAUS, but such a device is still in the development phase. Owing to the aging global population, we expect that the demand for an eAUS will be significant, so intensive research and development are necessary. Our survey results highlight the critical need for further investigation into potential technical malfunctions, patient concerns, and adaptation of this technology.

  ***Author contributions***: Marc Kidess had full access to all the data in the study and takes responsibility for the integrity of the data and the accuracy of the data analysis.

  *Study concept and design*: Kidess, Volz.

*Acquisition of data*: Kidess, Volz.

*Analysis and interpretation of data*: Kidess, Volz.

*Drafting of the manuscript*: Kidess, Volz.

*Critical revision of the manuscript for important intellectual content*: Stief, Ivanova, Stadelmeier, Hermans, Marcon, Bauer, Chaloupka, Pyrgidis, Hoffmann.

*Statistical analysis*: Volz.

*Obtaining funding*: None.

*Administrative, technical, or material support*: None.

*Supervision*: None.

*Other*: None.

  ***Financial disclosures:*** Marc Kidess certifies that all conflicts of interest, including specific financial interests and relationships and affiliations relevant to the subject matter or materials discussed in the manuscript (eg, employment/affiliation, grants or funding, consultancies, honoraria, stock ownership or options, expert testimony, royalties, or patents filed, received, or pending), are the following: None.

  ***Funding/Support and role of the sponsor*:** None.

## References

[1] Binder J., Kramer W. (2001). Robotically-assisted laparoscopic radical prostatectomy. BJU Int.

[2] Leow J.J., Chang S.L., Meyer C.P. (2016). Robot-assisted versus open radical prostatectomy: a contemporary analysis of an all-payer discharge database. Eur Urol.

[3] Rodler S., Kidess M.A., Westhofen T. (2023). A systematic review of new imaging technologies for robotic prostatectomy: from molecular imaging to augmented reality. J Clin Med.

[4] Schiavina R., Bianchi L., Lodi S. (2021). Real-time augmented reality three-dimensional guided robotic radical prostatectomy: preliminary experience and evaluation of the impact on surgical planning. Eur Urol Focus.

[5] Bauer R.M., Bastian P.J., Gozzi C. (2009). Postprostatectomy incontinence: all about diagnosis and management. Eur Urol.

[6] Haglind E., Carlsson S., Stranne J. (2015). Urinary incontinence and erectile dysfunction after robotic versus open radical prostatectomy: a prospective, controlled, nonrandomised trial. Eur Urol.

[7] Cornu J.N., Gacci M., Hashim H. (2024).

[8] Raj G.V., Peterson A.C., Toh K.L. (2005). Outcomes following revisions and secondary implantation of the artificial urinary sphincter. J Urol.

[9] Scott F.B., Bradley W.E., Timm G.W. (1973). Treatment of urinary incontinence by implantable prosthetic sphincter. Urology.

[10] Carson C.C. (2020). Artificial urinary sphincter: current status and future directions. Asian J Androl.

[11] Biardeau X., Hached S., Loutochin O. (2017). Montreal electronic artificial urinary sphincters: our futuristic alternatives to the AMS800™. Can Urol Assoc J.

[12] Shelli L. UroMems announces first-ever smart artificial urinary sphincter implant in a female patient (20.06.2023). https://www.prnewswire.com/news-releases/uromems-announces-first-ever-smart-artificial-urinary-sphincter-implant-in-a-female-patient-301882182.html.

[13] UroMems. Therapy. https://www.uromems.com/en/therapy.

[14] Beaugerie A., Mozer P., Poinard F. (2023). First in man implantation of the new artificial urinary sphincter UroActive (UroMems): preliminary results at 3 months post-activation follow-up of the first patient. Continence.

[15] Frazier R.L., Jones M.E., Hofer M.D. (2024). Artificial urinary sphincter complications: a narrative review. J Clin Med.

[16] Beaugerie A., Perrouin-Verbe M., Tran S. (2024). 137 – The new artificial urinary sphincter UroActive™ (UroMems): results of the first in man implantation study at 6 months post-activation (SOPHIA study). Continence.

[17] Kretschmer A., Buchner A., Grabbert M. (2016). Risk factors for artificial urinary sphincter failure. World J Urol.

[18] Assel M., Sjoberg D., Elders A. (2019). Guidelines for reporting of statistics for clinical research in urology. J Urol.

[19] Vakalopoulos I., Kampantais S., Laskaridis L. (2012). New artificial urinary sphincter devices in the treatment of male iatrogenic incontinence. Adv Urol.

[20] Gousse A.E., Madjar S., Lambert M.M. (2001). Artificial urinary sphincter for post-radical prostatectomy urinary incontinence: long-term subjective results. J Urol.

[21] Montague D.K., Angermeier K.W., Paolone D.R. (2001). Long-term continence and patient satisfaction after artificial sphincter implantation for urinary incontinence after prostatectomy. J Urol.

[22] Bertocchi F.M., de Oliveira A.C., Lucchetti G. (2022). Smartphone use, digital addiction and physical and mental health in community-dwelling older adults: a population-based survey. J Med Syst.

[23] Seifert A. (2022). Digitale Transformation in den Haushalten älterer Menschen. Z Gerontol Geriatr.

[24] Beaugerie A., Perrouin-Verbe M.A., Denormandie A. (2024). A0247 – A new automated electrical artificial urinary sphincter: results of the first in man study at 3 months post-activation (SOPHIA study). Eur Urol.

[25] Melkas H., Kohlbacher F., Herstatt C. (2011). The silver market phenomenon.

[26] Korchut A., Petit V., Szwedo-Brzozowska E. (2022). Assistive technology in multiple sclerosis patients-two points of view. J Clin Med.

[27] Ahmed O., Sallow A. (2017). Android security: a review. Acad J Nawroz Univ.

[28] Erharter D., Jungwirth B., Knoll B., Schwarz S., Posch P., Xharo E. (2014). Proceedings of Assitenztechnik für betreutes Wohnen: Beiträge zum Usability Day XII.

[29] Amann-Hechenberger B, Buchegger B, Erharter D, et al. Tablet & Smartphone: Seniorinnen und Senioren in der mobilen digitalen Welt. Research report for the mobi.senior.A project (www.mobiseniora.at); 2015. https://forschungsbericht.mobiseniora.at/forschungsbericht.pdf.

[30] Mostaghel R. (2016). Innovation and technology for the elderly: systematic literature review. J Bus Res.

[31] Kohlbacher F., Hang C.C. (2011). Applying the disruptive innovation framework to the silver market. Ageing Int.

