# Supplementary material for: Next-generation Solutions: Are Patients Ready for Electronic Artificial Urinary Sphincters for Male Incontinence?
Source: Eur Urol Open Sci. 2025 Feb 21;74:21–7. doi: 10.1016/j.euros.2025.02.004 (PMC11891701; doi:10.1016/j.euros.2025.02.004)
Supplement: Supplementary Data 1 [file mmc1.docx]

Dear patient,
Thank you very much for agreeing to support this survey by taking approx. 5 minutes to answer our questionnaire! We kindly ask you to answer the following questions truthfully. This survey is anonymous and no conclusions can be drawn about you personally. Please select **only one answer option** (🗷). If more than one answer is possible, this will be indicated separately.

1. How old are you?

☐ < 50 ☐ 50 – 60

☐ 61 – 70 ☐ 71 – 80

☐ 81 – 90 ☐ > 90

1. What is your highest level of education?

☐ Secondary School Leaving Certificate

☐ Intermediate School Leaving Certificate

☐ Higher School Certificate

☐ Vocational Training

☐ University Education

☐ Doctorate

☐ No Answer

1. What preconditions do you have? (multiple answers possible)

☐ Cardiovascular diseases (e.g. Hypertension)

☐ Neurological diseases (e.g. Parkinsons disease)

☐ Diabetes mellitus Type I/II

☐ Musculoskeletal diseases (e.g. Arthrosis)

☐ Oncological diseases (e.g. Prostate cancer)

☐ Others

☐ None

1. How many years ago did you receive the artificial urinary sphincter (AUS) implantation?

☐ < 1 ☐ 1-2

☐ 3-5 ☐ 6-10

☐ >10

1. Do you cope well with the manual handling of AUS?

☐ Yes ☐ No

1. Are you satisfied with the AUS?

☐ Yes ☐ No

1. Would you make the decision to haven an AUS again?

☐ Yes ☐ No

1. Which technical devices do you use regularly in everyday life? (multiple answers possible)

☐ Smartphone (e.g. iPhone)

☐ Tablet (e.g. iPad)

☐ Smart-Watch (e.g. Apple-Watch)

☐ Computer

☐ Others

1. Would you be open to having an eAUS implanted?

☐ Yes ☐ No

1. Would you like to operate this eAUS via an app on your cell phone?

☐ Yes ☐ No

1. Would you like to operate this eAUS via an app on your smartwatch?

☐ Yes ☐ No

1. Would you like to operate this eAUS via wireless (remote) control?

☐ Yes ☐ No

1. Would you like to be able to adjust the sphincter pressure electronically depending on the situation (sleep vs. sport)?

☐ Yes ☐ No

1. What would be your biggest concerns with an eAUS? (multiple answers possible)

☐ Failure of the battery

☐ Hacking of the operating app or the system

☐ Loss of connection between the control unit and the sphincter system

☐ Malfunction of the system

☐ Urethral injury

☐ Bleeding

☐ Infections

☐ Others

☐ None
